# Genetic Characterization of the Belgian Nephropathogenic Infectious Bronchitis Virus (NIBV) Reference Strain B1648

**DOI:** 10.3390/v7082827

**Published:** 2015-08-07

**Authors:** Vishwanatha R.A.P. Reddy, Sebastiaan Theuns, Inge D.M. Roukaerts, Mark Zeller, Jelle Matthijnssens, Hans J. Nauwynck

**Affiliations:** 1 Laboratory of Virology, Department of Virology, Parasitology and Immunology, Faculty of Veterinary Medicine, Ghent University, Salisburylaan 133, B-9820 Merelbeke, Belgium; E-Mails: Reddy.Vishwanatha@ugent.be (V.R.A.P.R.); Sebastiaan.Theuns@UGent.be (S.T.); inge.roukaerts@UGent.be (I.D.M.R.); 2 Rega Institute for Medical Research, Laboratory of Clinical Virology, Department of Microbiology and Immunology, KU Leuven—University of Leuven, Minderbroedersstraat 10, BE-3000 Leuven, Belgium; E-Mails: mark.zeller@uzleuven.be (M.Z.); jelle.matthijnssens@uzleuven.be (J.M.); 3 Rega Institute for Medical Research, Laboratory of Viral Metagenomics, Department of Microbiology and Immunology, KU Leuven—University of Leuven, Minderbroedersstraat 10, BE-3000 Leuven, Belgium

**Keywords:** IBV, B1648, nephropathogenic, chicken, nonstructural protein

## Abstract

The virulent nephropathogenic infectious bronchitis virus (NIBV) strain B1648 was first isolated in 1984, in Flanders, Belgium. Despite intensive vaccination, B1648 and its variants are still circulating in Europe and North Africa. Here, the full-length genome of this Belgian NIBV reference strain was determined by next generation sequencing (NGS) to understand its evolutionary relationship with other IBV strains, and to identify possible genetic factors that may be associated with the nephropathogenicity. Thirteen open reading frames (ORFs) were predicted in the B1648 strain (5′UTR-1a-1b-S-3a-3b-E-M-4b-4c-5a-5b-N-6b-3′UTR). ORFs 4b, 4c and 6b, which have been rarely reported in literature, were present in B1648 and most of the other IBV complete genomes. According to phylogenetic analysis of the full-length genome, replicase transcriptase complex, spike protein, partial S1 gene and M protein, B1648 strain clustered with the non-Massachusetts type strains NGA/A116E7/2006, UKr 27-11, QX-like ITA/90254/2005, QX-like CK/SWE/0658946/10, TN20/00, RF-27/99, RF/06/2007 and SLO/266/05. Based on the partial S1 fragment, B1648 clustered with the strains TN20/00, RF-27/99, RF/06/2007 and SLO/266/05 and, further designated as B1648 genotype. The full-length genome of B1648 shared the highest sequence homology with UKr 27-11, Gray, JMK, and NGA/A116E7/2006 (91.2% to 91.6%) and was least related with the reference Beaudette and Massachusetts strains (89.7%). Nucleotide and amino acid sequence analyses indicated that B1648 strain may have played an important role in the evolution of IBV in Europe and North Africa. Further, the nephropathogenicity determinants might be located on the 1a, spike, M and accessory proteins (3a, 3b, 4b, 4c, 5a, 5b and 6b). Overall, strain B1648 is distinct from all the strains reported so far in Europe and other parts of the world.

## 1. Introduction

Infectious bronchitis virus (IBV) belongs to the *Nidovirales*, family *Coronaviridae,* subfamily *Coronavirinae* and genus *Gammacoronavirus*. Coronaviruses of turkeys, ducks, pheasants, teal and geese, as well as Beluga whale and Bottlenose dolphin coronaviruses are other known members within this genus [1,2]. IBV is an enveloped virus with a positive sense single stranded RNA genome. The genome has a size of approximately 27.6 kb and contains a methylated cap and poly (A) tail at its 5′ and 3′ end, respectively. IBV contains at least ten open reading frames (ORFs) in the order 5′ UTR-1a-1b-S-3a-3b-EM-5a-5b-N-3′UTR [3]. Gene 1 or the replicase transcriptase complex gene is the largest gene (20 kb) among the ten ORFs. Gene 1 consists of two overlapping ORFs, namely ORF1a and ORF1b, of which the latter is translated in polyprotein 1ab by a ribosomal frameshift. Furthermore, ORF1a and ORF1b encode 15 non-structural proteins (NSPs), which are required for RNA replication, transcription and other aspects of viral replication and pathogenesis. ORFs 2, 3, 4 and 6 encode four major structural proteins: The spike (S) glycoprotein, the small envelope (E) protein, the membrane (M) glycoprotein and the nucleocapsid (N) protein, respectively. The S protein is cleaved into two subunits, namely S1 and S2, of which S1 is the most variable domain and a major serotype determinant. ORFs 3 and 5 are interspersed between ORFs encoding structural proteins, and encode small non-structural proteins (NSPs), known as 3a, 3b, 5a and 5b [3]. Overall, the IBV genome is genetically variable due to the frequent occurrence of point mutations, insertions, deletions and recombination events [3,4,5].

IBV affects both broiler and layer chickens. Although chicken flocks are routinely vaccinated with live vaccines, outbreaks of infectious bronchitis have been observed in vaccinated flocks, as there is little or no cross-protection between different IBV serotypes. Hence, serological and molecular characterization of field isolates is very important to select appropriate vaccine strains. IBV has a tropism for the epithelial cells of the respiratory tract, kidney, oviduct and alimentary tract of chickens. In the beginning of the 1950s, the respiratory Massachusetts (Mass) type of IBV was identified in the United States. Later, Mass-type strains have been isolated all over the world and variants emerged. In Europe, at present, Mass 41, 4/91, D274, Italy 02 and QX are recognized as important circulating serotypes [3,6]. Some IBV strains were described as nephropathogenic since the respiratory infection was followed by a severe renal infection, which leads to clinical signs such as excessive water consumption and wet droppings and increased mortality. Post-mortem examination of birds that died after a nephropathogenic IBV (NIBV) infection reveals dehydrated carcasses and swollen and pale kidneys with urates in the tubules.

The first nephropathogenic IBV strains were reported in the US and Australia, and later in other parts of the world [7,8,9,10,11]. Over the past 15 years, the nephropathogenic IBV strains have been emerging as most predominant IBV strains in poultry industry, especially in Asian and Middle Eastern countries [12,13,14,15,16]. Strain B1648 was responsible for outbreaks of kidney disease on chicken farms in Belgium, The Netherlands and Northern France, and was first isolated in 1984 [6,7,17]. At present, strain B1648 or its variants are still circulating in Europe and North Africa [18,19,20,21,22,23]. Based on the spike gene analysis of B1648 strain, some of the European, American and West African non-Massachusetts type strains show close genetic relationship with strain B1648 [19,24]. However, conclusions based on the spike gene or partial spike gene segment analysis should be made cautiously, because the true evolutionary relationship of B1648 with other strains can only be evaluated by complete genome analysis. Hence, this paper aims to characterize the complete genome of strain B1648 by means of NGS (Illumina) and aimed to identify the genetic factors, which may be associated with the nephropathogenic nature of this strain.

## 2. Materials and Methods

### 2.1. Virus Propagation

The virulent nephropathogenic IBV strain, B1648 has been isolated in 1984 [17]. Virus stocks were prepared in 10-day-old embryonated SPF chicken eggs by allantoic route inoculation. Then, virus was propagated in embryos for 48 h at 37 °C. Finally, the allantoic fluid was harvested, clarified by low speed centrifugation and frozen at −70 °C until use.

### 2.2. Preparation of RNA for Illumina Sequencing

The virus infected allantoic fluid was filtered twice using 0.8 μm and 0.45 μm membrane filters. Free and bacterial DNA/RNA was destroyed by the addition of 2 μL of Benzonase Nuclease (Novagen, San Diego, CA, USA), 1 μL of Micrococcal Nuclease (New England Biolabs, Ipswich, MA, USA) and 1 μL of NEBNext^®^ RNase III RNA Fragmentation Module (Invitrogen, Carlsbad, CA, USA) in 7 μL of homemade buffer (1 M Tris, 100 mM CaCl_2_ and 30 mM MgCl_2_) that were added to 140 μL of allantoic fluid, and incubated for 2 h at 37 °C. Next, 7 μL of EDTA were added to the sample for enzyme inactivation. Extraction of viral RNA was performed using the QIAamp Viral RNA Mini Kit (Qiagen, Hilden, Germany) according to the manufacturer’s instructions, but without using carrier RNA. Total RNA was amplified using the Whole Transcriptome Amplification Kit (WTA 2, Sigma Aldrich, St. Louis, MO, USA). Therefore, 0.5 μL Library Synthesis Solution was added to 2.82 μL of RNA, followed by denaturation for 2 min at 95 °C. RNA was cooled to 18 °C and 0.5 μL Library Synthesis Buffer, 0.4 μL Library Synthesis Enzyme and 0.78 μL of water was immediately added to the reaction. The mixture was treated with the following temperature conditions: 18 °C, 25 °C, 37 °C, 42 °C and 70 °C for 10, 10, 30, 10 and 20 min, respectively. Samples were cooled down to 4 °C followed by a brief centrifugation step. A mastermix containing 60.2 μL of nuclease free water, 7.5 μL of Amplification Mix, 1.5 μL of WTA dNTP mix and 0.75 μL Amplification Enzyme was added to the sample and incubated as follows: 94 °C for 2 min and 30 cycles at 94 °C for 30 s and 70 °C for 5 min. The resulting cDNA products were purified with the MSB^®^ Spin PCRapace kit (STRATEC Molecular, Birkenfeld, Germany) according to the instructions of the manufacturer and prepared for Illumina sequencing using the KAPA Library Preparation Kit (Kapa Biosystems, Wilmington, NC, USA), according to the manufacturer’s instructions.

### 2.3. Illumina Sequencing and Sequence Assembly

Fragments ranging from 350–600 bp were selected using the BluePippin (Sage Science, Beverly, MA, USA) according to the manufacturer’s instructions. Sequencing of the samples was performed on a HiSeq 2500 platform (Illumina, San Diego, CA, USA) for 300 cycles (150 bp paired ends). Raw reads were trimmed for quality and adapters, and were *de novo* assembled into contigs using SPAdes [25]. Scaffolds were classified using a tBLASTx search against all complete viral genomes in GenBank using an *e-*value cut-off of 10^−10^. Scaffolds with a significant tBLASTx hit were retained and used for a second tBlastx search against the GenBank nucleotide database using an E-value of 10^−4^ [26].

### 2.4. Genome Sequence Analysis

Multiple sequence alignments were performed using the ClustalW plug-in in the MEGA software version 5.2.2, followed by manual editing. The B1648 genome, coding sequence and ORF prediction was carried out in http://covdb.microbiology.hku.hk and http://www.jcvi.org/vigor/ [27,28]. Phylogenetic trees were constructed using the maximum-likelihood method. Substitution models were determined for each gene separately. The bootstrap values were determined from 500 replicates of the original data. Nucleotide and amino acid identities were determined using the p-distance model. The complete genome of strain B1648 was compared to 55 relevant respiratory and nephropathogenic complete IBV genomes of North America (USA), Asia (China, Korea and Taiwan), Africa (Nigeria) and Europe (Sweden, Ukraine and Italy). Partial S1 gene sequences (727 nt) of 90 relevant respiratory and nephropathogenic IBV strains of North America (USA), South America (Argentina), Europe (Sweden, Italy, UK, Slovenia, Ukraine and Russia), Asia (China, India, Israel, Korea and Taiwan), Africa (Tunisia, Nigeria and Egypt) and Australia were compared to B1648 strain as well.

### 2.5. Recombination Analysis

Simplot analysis (SimPlot version 3.5.1) was performed to determine whether the B1648 strain has recombined with other strains during its evolution. Based on the phylogenetic analysis of the complete genome sequence, 10 relevant strains were selected and included in the recombination analysis. The 10 complete genome sequences were aligned using the ClustalW plug-in in the MEGA software version 5.2.2. The Kimura 2-parameter model was used as a distance model, the window size was 500 bp and step size was 60 bp.

### 2.6. GenBank Accession Number

The full-length genomic sequence of the B1648 strain that was described in this report has been deposited in the GenBank database with accession number KR231009.

## 3. Results

### 3.1. Genome Organization of Strain B1648

The complete genome sequence of strain B1648 had a size of 27654 nucleotides (nt), excluding the poly(A)-tail. Thirteen ORFs (5′-1a-1b-S-3a-3b-E-M-4b-4c-5a-5b-N-6b-3′) were predicted in the B1648 genome (Table 1). ORFs 4b, 4c and 6b were predicted in the B1648 genome and also in most of the GenBank IBV genomes (Table 2). Among the different regions of the genome, the 5′ end untranslated regions (UTR) (518 nt) and 3′ end UTR (292 nt) were most conserved (94.8% to 99.5%). On the other hand, the 6b protein was the most variable (15.9% to 96%), followed by 4c (46.4% to 100%), 3b (51.2% to 96.4%) and the Spike protein (56.9% to 86.2%) (Table 2). Deletions, insertions and point mutations were distributed throughout the B1648 genome.

**Table 1:** Genes, coding regions, nucleotide length and amino acids size of B1648 strain.

Open Reading Frame	Frame	Nucleotide Location	Nucleotide Length (bp)	Amino Acids Size
5′ UTR	-	1–518	518	-
1a	+3	519–12368	11,850	3949
1b	+2	12443–20401	7959	2652
Spike	+3	20352–23852	3501	1166
3a	+2	23852–24025	174	57
3b	+1	24025–24219	195	64
Envelope	+2	24200–24484	285	94
Membrane	+3	24477–25154	678	225
4b	+3	25155–25439	285	94
4c	+1	25360–25530	171	56
5a	+2	25514–25711	198	65
5b	+1	25708–25956	249	82
Nucleocapsid	+3	25899–27128	1230	409
6b	+2	27137–27361	225	74
3′ UTR	-	27362–27654	292	-

**Table 2:** Nucleotide and amino acid sequence identity (%) of B1648 genome with relevant genomes of Infectious Bronchitis Virus (IBV).

Strain	Genome	5′	1a	1b	Spike	3a	3b	E (3c)	M	4b	4c	5a	5b	N	6b	3′
Beaudette	89.7	96.2	89.7/91.7	91.9/96.6	80.7/80.7	91.1/94.6	80.1/64.6	88.9/81.8	95.1/93	90.2/85.8	87.1/84.2	83.4/84.8	95.3/90.8	92.4/93.6	93.6/93.9	**99.1**
California99	90.4	98.1	90.1/91.9	93/97.1	79.2/**84.1**	87.8/82.8	96.6/96.4	92.3/90.7	93.9/91.5	89.6/87.5	89.0/89.7	86.5/88.4	93.6/86.6	93.4/96	68.9/65.2	98.1
Cal5572003	90.4	98.4	90.3/91.3	92.9/97.2	79.5/83	93.7/94.6	89.8/80.7	93.5/91.9	94.4/91.5	83.7/76.9	64.4/50.5	87.1/86.6	94.4/86.6	93.3/94.7	94.3/96	98.1
Cal56b	90.6	97.8	90.7/92.1	92.9/97	77.8/82.8	89.2/85.9	94.8/92.7	91.5/88.2	95.1/93	85.5/82.4	66.0/50.5	88.3/88.4	94.5/90.8	94.1/96.2	93/93.9	98.6
SAIBK	86.8	95.8	86.4/89.4	*89.5/95.5*	78/81.7	90.9/79.6	76.2/67.1	88.5/85.7	92/90.4	*83/82.4*	*64.4/46.4*	87.2/91.9	93.6/89.5	85.7/91.8	NA	97.6
TW2575/98	*86.0*	*95.5*	*84.8/87.9*	*88.9/95.7*	79.2/81.7	94.8/**97.3**	87.3/80.7	90.5/87	92.7/90.4	83.7/80.6	85.1/81.3	86.3/88.4	92.6/85.2	88.4/92	68.3/65.2	97.1
ArkDPI11	90.8	98.7	90.6/92.2	93/96.9	80.7/83.7	97.4/94.6	96.6/94.6	**93/91.9**	93.6/92	89.6/87.5	89.9/92.4	86.5/88.4	94.9/89.5	94.2/96.5	94.3/96	98.1
H52	89.6	97.8	89.4/91.5	92.1/96.5	80.9/80.6	85.1/88.9	81.5/67.1	88.9/81.8	95.4/93	88.5/85.8	85.2/78.3	87.8/88.4	92.6/85.2	91.9/94.7	*34.7/15.9*	98.6
H120	90.4	**98.7**	89.9/91.7	**93.6/97.4**	79/81.1	85.1/88.9	80.7/67.1	87.6/76.3	96.2/92.5	88.5/85.8	85.2/78.3	88.4/88.4	95.3/90.8	93.4/94.7	*34.7/15.9*	99.0
Mass412006	90.8	**98.7**	91.1/92.6	92.5/97	83.1/82.3	87.8/82.8	96.6/96.4	93.5/**93.1**	93.7/92	89.6/87.5	89.9/92.4	86.5/88.4	94.4/88.1	**94.4/96.7**	93/93.9	98.6
Mass411985	89.7	96.8	89.6/91.4	92.2/96.7	*58.3*/80.8	91.1/91.8	81.5/67.1	89.5/83.1	94.8/93	90.9/89.1	89.2/81.3	86.6/83	*92.6/83.7*	91.9/94.7	NA	98.1
Conn461996	91.1	97.8	91.5/92.7	93.1/97	58.6/82.5	87.8/82.8	96.6/96.4	93.3/93.1	93.6/92	89.6/87.5	89.9/92.4	86.5/88.4	94.9/89.5	**94.4/96.7**	89.5/91.8	98.6
ITA/90254/2005	90.2	97.1	**92.2/94.2**	90.8/96.3	80.5/81.8	85.7/79.6	*64.1/54*	89.2/84.4	95.6/94.6	93.4/92.4	92.7/89.7	90.1/91.9	92.6/86.6	91.3/94.9	97.5/96	99.0
NGA/A116E7/2006	**91.6**	97.8	**92.2/94**	93/96.8	81.7/**84.8**	90/79.6	78.1/64.6	92/90.7	89.9/85.5	92.4/90.8	91.9/89.7	93.5/91.9	93.5/88.1	92.8/95.2	94.2/89.7	98.6
Delaware072	88.1	98.4	89.1/90.5	*91/94.2*	81.4/57.5	92.8/82.8	96.6/96.4	92.9/90.7	94.7/93	88.5/85.8	85.1/78.3	85.3/88.4	94.9/89.5	92.9/94.4	92.9/93.9	98.6
Gray	**91.2**	98.7	91.8/93.3	92.6/97.2	78.6/**82.8**	97.4/94.6	96.6/94.6	92.6/91.9	*88.2/81.6*	89.6/85.8	89.9/92.4	86.5/88.4	93.1/86.6	*86.1/84.5*	94.9/96	*96.6*
Holte	90.7	98.7	91.5/92.8	92.2/97	*79/80.2*	91.9/88.9	94.2/88.9	91.4/89.5	94.9/92	87.9/82.4	89.1/84.2	*83.3/86.6*	**95.8/92.2**	91.2/93.4	NA	NA
Iowa97	90.6	98.4	91.3/92.4	92.2/97	77.7/82.7	91.9/88.9	94.2/88.9	91.4/89.5	94.5/91.5	87.9/82.4	89.1/84.2	*83.3/86.6*	**95.8/92.2**	91.5/93.9	91.5/89.7	96.6
JMK	**91.2**	98.7	92/93.5	92.5/97	79.1/82.3	97.4/94.6	96/92.7	92.6/91.9	94.1/92	89.6/85.8	89.9/92.4	86.5/88.4	93.1/86.6	93.6/94.9	NA	98.1
CK/CH/LDL/101212	90.4	98.4	89.8/91.6	**93.6/97.3**	78.8/81.2	85.1/88.9	80.7/67.1	*87.6/76.3*	96.2/92.5	88.5/85.8	85.2/78.3	87.8/86.6	95.3/90.8	93.2/94.4	34.7/15.9	98.1
CK/SWE/0658946/10	89.5	96.8	90/91.1	91.3/95.8	80/80.8	*83.6*/76.4	*64.9/51.2*	**93.7/**88.2	96.1/95.6	91.3/92.4	92.0/81.3	**94.7/98.4**	91.2/85.2	89.2/90.4	97.5/96	99.0
KM91	90.0	98.7	91.1/92.6	91.7/96.6	*62.4/80.4*	87.8/69.5	78.8/74.1	89.6/89.5	94.3/93.5	92.5/92.4	93.6/95.0	86/84.8	93.1/86.6	92.5/95.5	71.4/73.2	96.6
CK/CH/LJL/111054	90.7	98.7	90.2/92.1	93.6/97.3	84.6**/**82.6	87.8/82.8	96.6/96.4	93.3/93.1	93.7/92	89.6/87.5	89.9/92.4	86.5/88.4	94.9/89.5	94.4/96.7	90.2/91.8	98.6
Ukr27-11	**91.2**	96.5	89.8/91.7	**93.6/97.2**	**80.8/86.2**	**98.3/97.3**	93/92.7	92.3/91.9	**96.9/96.6**	**97.6/97**	**96.9/100.0**	94.7/93.5	94.4/89.5	93.2/**95.2**	**97.5/96**	99.0
CK/CH/LDL/110931	90.6	98.7	89.9/91.7	93.6/97.3	80.7/82.4	87.8/82.8	**96.6/96.4**	93.3/93.1	93.7/92	89.6/87.5	89.9/92.4	86.5/88.4	94.9/89.5	**94.4**/96.7	90.2/91.8	98.6

Boldface indicates the highest, and italic the lowest, nucleotide and amino acid sequence identity.

### 3.2. Phylogenetic Analysis and Comparison Alignments of Full Genomes of IBV Strains

**Figure 1:**
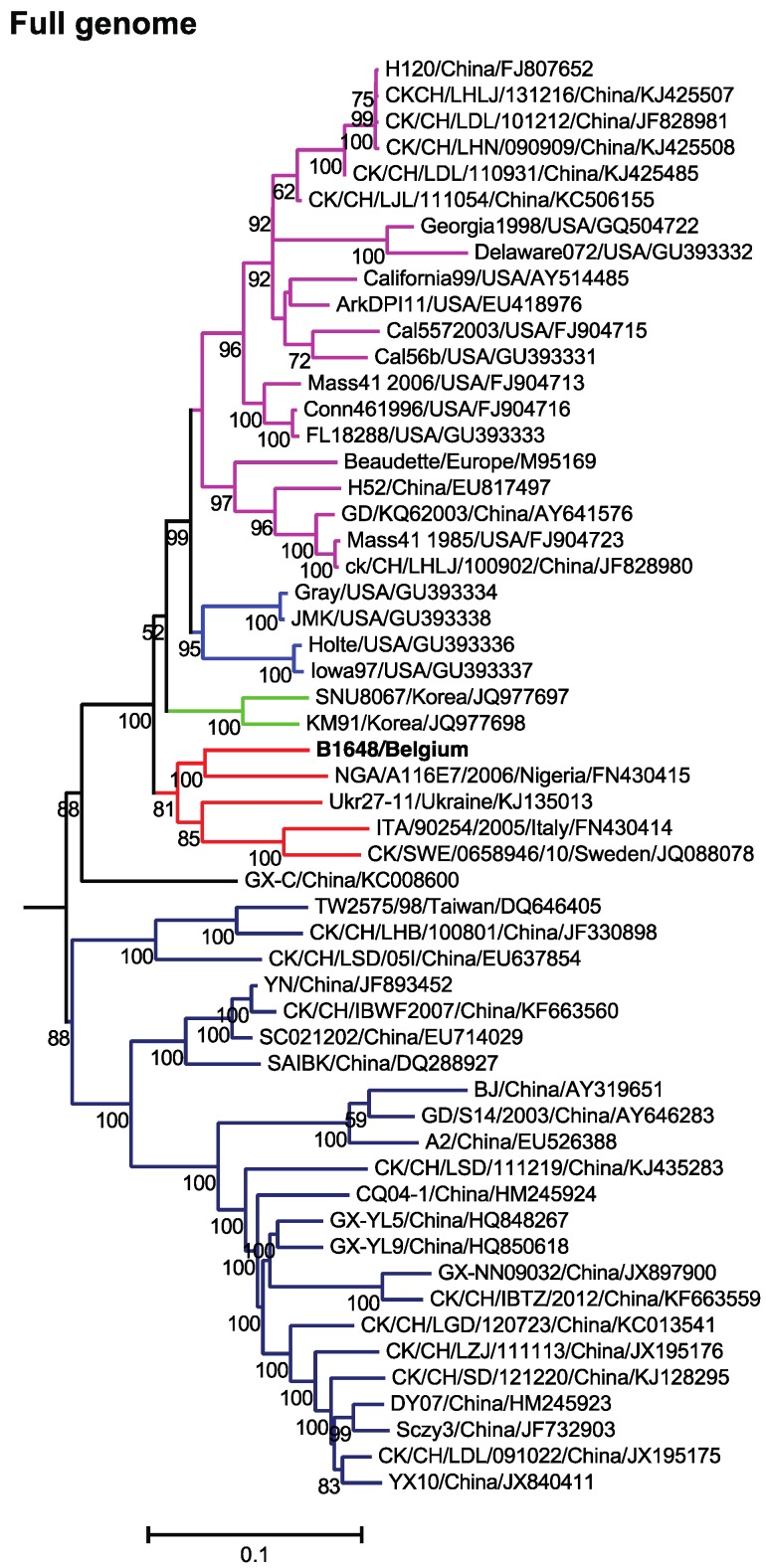
Maximum likelihood phylogenetic tree based on 55 full genome sequences. Bootstrap values (*n* = 500 replicates) of <50% are not shown. Strain B1648 cluster is shown in red.

The general time reversible model with gamma distribution and invariant sites was used for the construction of a maximum likelihood phylogenetic tree of the 55 relevant respiratory and nephropathogenic complete IBV genomes of North America (USA), Asia (China, Korea and Taiwan), Africa (Nigeria) and Europe (Sweden, Ukraine and Italy) (Figure 1). The phylogenetic analysis of 55 full-length nucleotide sequences of different IBV strains has demonstrated that NGA/A116E7/2006 (non-Mass pathogenic variable type of Nigeria), UKr 27-11 (non-Mass recombinant type of Ukraine), QX-like ITA/90254/2005 (non-Mass type of Italy) and QX-like CK/SWE/0658946/10 (non-Mass type of Sweden) strains clustered most closely with B1648.

The B1648 strain has the highest nucleotide sequence homology with strains UKr 27-11, Gray (non-Mass nephropathogen), JMK (non-Mass respiratory pathogen) and NGA/A116E7/2006 (91.2% to 91.6%) (Table 2). Strain B1648 was clustered distinctly from all the Mass type strains. The nucleotide sequence homology to Mass 41 (M41, 1985) and reference (Beaudette) strain was 89.7%.

### 3.3. Phylogenetic Analysis and Sequence Comparison of Spike Protein (1166 aa) and Partial S1 Gene (727 nt)

**Figure 2:**
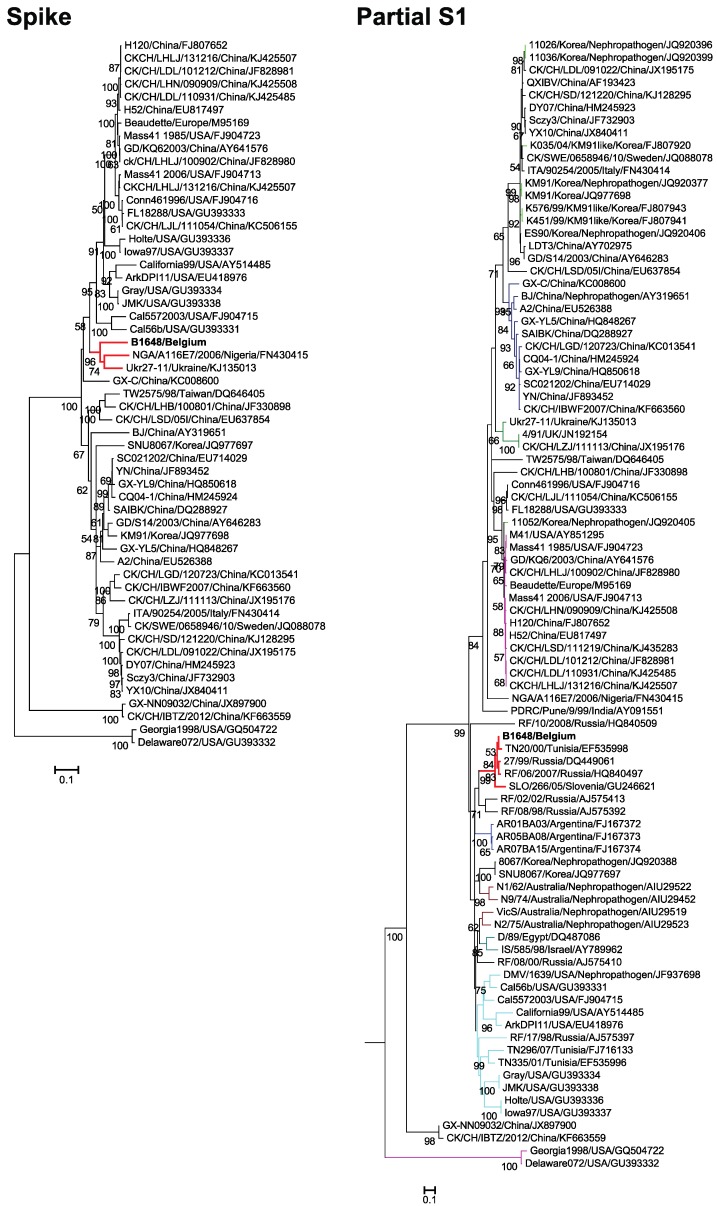
Maximum likelihood phylogenetic trees based on amino acid sequences of spike protein and nucleotide sequences of partial S1 gene. Bootstrap values (*n* = 500 replicates) of <50% are not shown. Strain B1648 cluster is shown in red.

Maximum likelihood trees were constructed for the amino acid sequences of the spike protein and nucleotide sequences of the partial S1 gene using the general time reversible and Tamura 92 models with gamma distribution sites, respectively (Figure 2). The phylogenetic analysis of the spike protein (1166 aa) clustered NGA/A116E7/2006 and Ukr27-11 with B1648 strain. The phylogenetic analysis of 90 partial S1 genes (727 nt) of relevant respiratory and nephropathogenic IBV strains of North America (USA), South America (Argentina), Europe (Sweden, Italy, UK, Slovenia, Ukraine and Russia), Asia (China, India, Israel, Korea and Taiwan), Africa (Tunisia, Nigeria and Egypt) and Australia has clustered B1648 strain with TN20/00 (Tunisian), RF-27/99 and RF/06/2007 (Russian) and SLO/266/05 (Slovenian) strains.

The Spike protein of B1648 showed the highest amino acid identities to California99, NGA/A116E7/2006 and Ukr27-11 (84.1% to 86.2%). The partial S1 gene of B1648 strain showed the highest nucleotide homology with strains SLO/266/05, RF/06/2007, RF-27/99 and TN20/00 (89.4% to 97.4%). According to amino acid sequence identity, the Spike protein (56.9% to 86.2%) was the fourth most variable region (Table 2).

### 3.4. Phylogenetic Analysis and Comparison Alignments of the Replicase Transcriptase Complex (Polyprotein 1a (3949 aa) and 1b (2652 aa))

**Figure 3:**
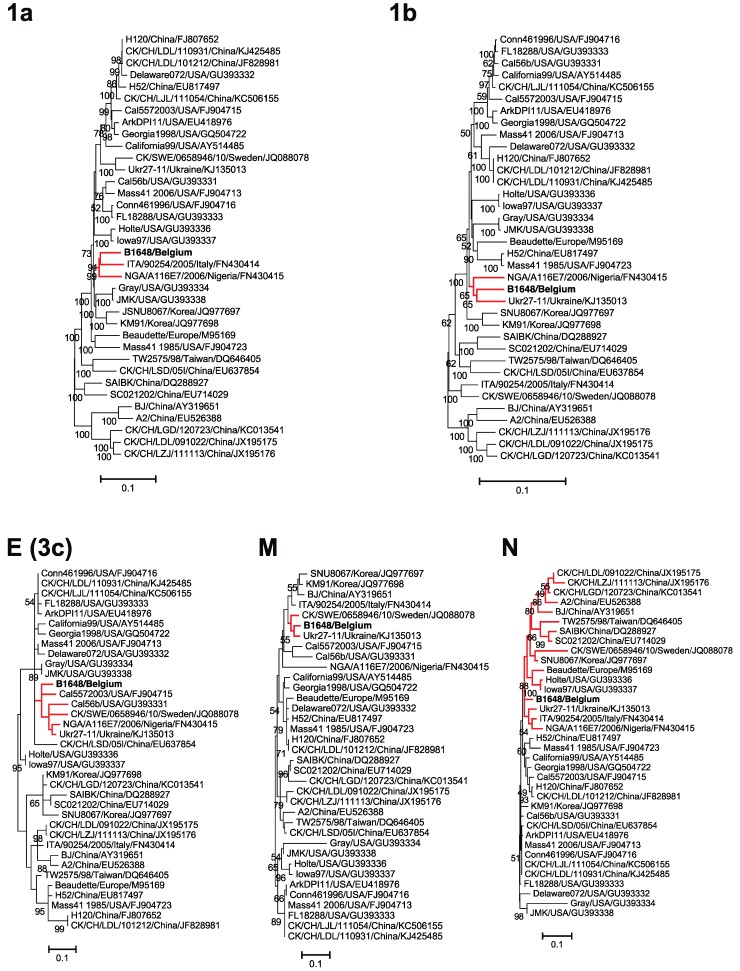
Maximum likelihood phylogenetic trees based on amino acid sequences of 1a, 1b, E (3c), M and N proteins. Bootstrap values (*n* = 500 replicates) of <50% are not shown. Strain B1648 cluster is shown in red.

The replicase transcriptase complex (polyprotein 1a (3949 aa) and 1b (2652 aa)) maximum likelihood trees were constructed using the general time reversible model with gamma distribution and invariant sites (Figure 3). Based on the phylogenetic analysis of the replicase protein complex amino acid sequences, B1648 clustered with NGA/A116E7/2006 (1a, 1b), ITA/90254/2005 (1a) and UKr 27-11 (1b).

Polyprotein 1a (3949 aa) had the highest amino acid homology with strains Gray, NGA/A116E7/2006 and ITA/90254/2005 (93.3% to 94.2%), while polyprotein 1b (2652 aa) was closest to CK/CH/LDL/101212, CK/CH/LHLJ/131216 and H120 (97.3% to 97.4%). Overall, polyprotein 1b (95.3% to 97.4%) was more conserved than 1a (84.8% to 94.6%) (Table 2). Among the 15 NSPs of polyprotein 1ab, NSP3 (80.6% to 92.4%), NSP9 (69.1% to 99.1%) and NSP11 (69.8% to 86%) were most variable, whereas the other NSPs were generally more conserved (82.8% to 100%) (Supplementary Table S1).

### 3.5. Phylogenetic Analysis and Sequence Comparison of Amino Acid Sequences of E, M and N Proteins

**Figure 4:**
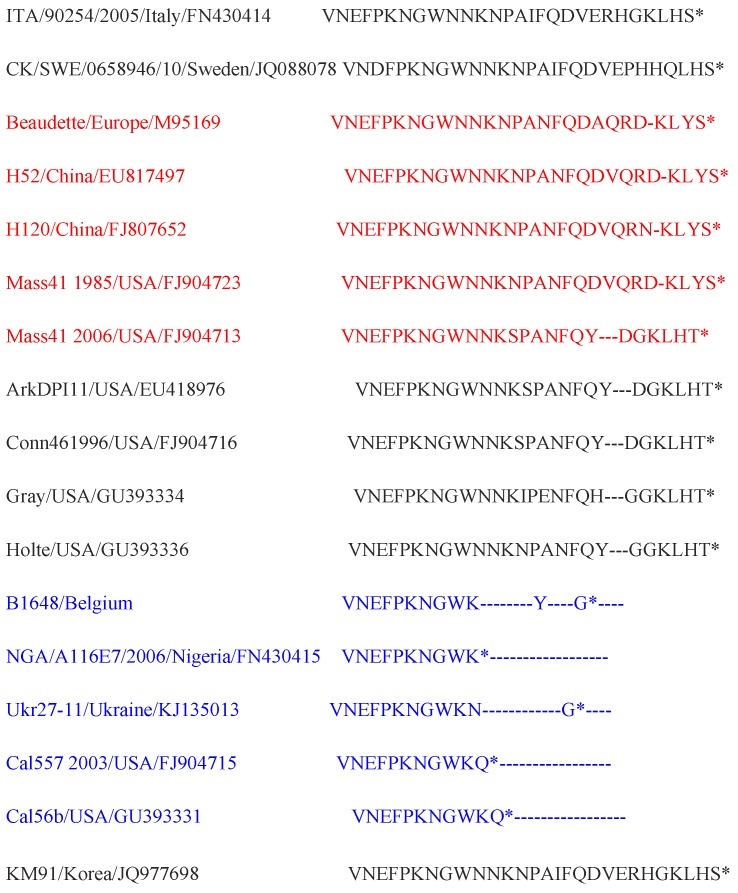
Amino acid sequence differences in C-terminal of E protein of B1648 strain with other Mass (red) and non-Mass type strains. In the B1648 strain 3′ terminal E (3c) protein, a region of total 12 amino acids was discontinuously deleted (blue). The dashes (-) indicate the deleted sequences.

For construction of E or 3c (94 aa), M (225 aa) and N (409 aa) proteins maximum likelihood trees, the Jones Thornton Taylor model with gamma distribution sites was used (Figure 3). Based on the phylogenetic analysis of amino acid sequences of E protein, NGA/A116E7/2006, Ukr27-11, Cal5572003, Cal56b and many other Mass and non-Mass type strains were clustered together with B1648. Further, a sequence of 12 amino acids (36 nucleotides) was discontinuously deleted in the C-terminal of the B1648 E protein. The same 12 amino acids were deleted in strains NGA/A116E7/2006, Ukr27-11, Cal5572003 and Cal56b (Figure 4). According to amino acid sequences of the M protein, B1648 strain was clustered with NGA/A116E7/2006, UKr 27-11, QX-like ITA/90254/2005, QX-like CK/SWE/0658946/10 and many other non-Mass type strains. Based on the amino acid sequences of N protein, NGA/A116E7/2006, UKr 27-11, QX-like ITA/90254/2005, Beaudette, Mass type (1985) and many other Mass type and non-Mass type strains were clustered together with B1648.

The E or 3c (94 aa) protein had the highest amino acid identities to Mass 41(2006), Conn461996, CK/CH/LDL/110931 and CK/CH/LJL/111054 (93.1%). The M protein (225 aa) was closest to ITA/90254/2005, CK/SWE/0658946/10 and Ukr27-11 (94.6% to 96.6%). The N protein (409 aa) showed the highest amino acid identities to Mass 41(2006), and many other Mass and non-Mass type strains (96.7%).

### 3.6. Accessory Proteins Alignments

There were 7 probable accessory proteins in the B1648 strain, such as 3a (57 aa), 3b (64 aa), 4b (94 aa), 4c (56 aa) 5a (65 aa), 5b (82 aa) and 6b (74 aa). The accessory proteins 3a (69.5% to 97.3%), 3b (51.2% to 96.4%), 4b (76.9% to 97%), 4c (46.4% to 100%) and 6b (15.9% to 96%) were variable whereas 5a (81.1% to 93.5%) and 5b (82.3% to 92.2%) were more conserved (Table 2). The highest amino acid identities of 3a, 3b, 4b, 4c, 5a, 5b and 6b were 97.3% (Ukr27-11 and TW2575/98), 96.4% (CK/CH/LDL/110931, California99 and many other non-Mass type strains), 97% (Ukr27-11), 100% (Ukr27-11), 98.4% (CK/SWE/0658946/10), 92.2% (Holte and Iowa97) and 96% (Ukr27-11, CK/SWE/0658946/10, Gray, ArkDPI11 and many other non-Mass type strains), respectively.

### 3.7. Recombination Analysis

**Figure 5:**
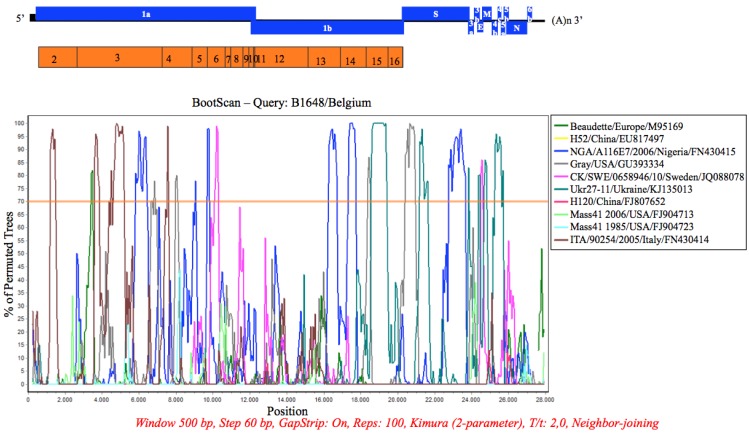
Simplot analysis (Simplot 3.5.1) of B1648 strain. The full genome sequence of B1648 strain was tested against 10 relevant IBV strains. The 70% bootstrap level is considered.

B1648 was used as a putative parental strain and 10 relevant pathogenic and vaccine strains were queried in the Simplot analysis (Figure 5). The B1648 strain was considered as parental strain, because the strains that clustered with B1648 (Figure 1, Figure 2 and Figure 3), were reported after the B1648 outbreak. In gene 1a, a part of NSP2 and NSP4 showed a higher similarity to ITA/90254/2005; a part of NSP3 showed a higher similarity to ITA/90254/2005 and NGA/A116E7/2006, and part of NSP6 showed a higher similarity to CK/SWE/0658946/10. In the gene 1b, a part of NSP13 and NSP14 shared a higher similarity with NGA/A116E7/2006, and a part of NSP15 did that with UKr 27-11. In the S gene, a part of the S1 region showed similarities with Gray and UKr 27-11 and a part of S2 did that with NGA/A116E7/2006. The 4b, 4c and 5a genes were very similar to those of UKr 27-11.

## 4. Discussion

The B1648 strain is a Belgian reference nephropathogenic IBV serotype. Earlier, in our laboratory it was demonstrated that B1648 was antigenically different from the Mass type and other variant strains. Furthermore, it was shown that Mass type vaccines (H120 and D274) did not provide protection [17,29]. More recently, Cook *et al.* [30] found that the 4/91 type (variant type) vaccine alone or the 4/91 and Ma5 (Mass type) combination vaccine protected against B1648 nephropathogenicity. In spite of the intensive vaccination program, the B1648 strain or its variants are still circulating or reemerging throughout Europe and North Africa [18,19,20,21,22,23]. Hence, in the present study the complete genome of the B1648 strain was characterized to identify putative genetic factors that may be involved in the tissue tropism, and to understand their role in evolution [7,17,29].

The B1648 genome organization (5′-1a-1b-S-3a-3b-E-M-4b-4c-5a-5b-N-6b-3′) was slightly different from most previously reported IBV genomes (5′-1a-1b-S-3a-3b-E-M-5a-5b-N-3′). 4b, 4c and 6b were additional ORFs present in the B1648 genome. Although 4b, 4c and 6b were present in the most of the IBV genomes, they have rarely been reported in literature [31,32,33]. ORFs 4b, 4c and 6b have also been reported in a turkey coronavirus [34]. The exact reason for the rare reports of these ORFs (4b, 4c and 6b) in most of the IBV genomes is not known. It could be that 4b, 4c and 6b ORFs are present in most of the IBV genomes, but that the success of their identification depends on the algorithms of ORF prediction software that was used. Recently, Bentley *et al.* [35] has demonstrated and confirmed 4b as a 5th accessory protein in IBV, besides 3a, 3b, 5a and 5b. The 4b homologue of Middle East respiratory syndrome coronavirus (MERS-CoV) was found to be an interferon antagonist [36]. The 6b homologue of SARS coronavirus was found to induce apoptosis [37]. For IBV, further investigations are necessary to demonstrate the production of 4b, 4c and 6b proteins and to identify their functions in pathogenesis.

The phylogenetic analysis of the full-length genome, replicase transcriptase complex, spike protein, partial S1 gene and M protein has suggested that B1648 may have played an important role in evolution, because the strains which were clustered (NGA/A116E7/2006, UKr 27-11, QX-like ITA/90254/2005, QX-like CK/SWE/0658946/10, TN20/00, RF-27/99, RF/06/2007 and SLO/266/05) with B1648 were reported in Europe and North Africa, after the initial B1648 outbreak. The geographical proximity of all these countries and frequent movements of poultry and their products in between these countries might be an important reason for this observed cluster. All the above clustered strains belong to a group referred to as non-Mass type strains. In the above-mentioned cluster, QX like ITA/90254/2005 and QX like CK/SWE/0658946/10 are presently predominant IBV strains in Europe (Germany, The Netherlands, Belgium, France, Sweden, Poland, Russia, Slovenia, Spain and the UK) [9,38,39,40,41,42,43,44]. The QX IBV strains were first reported in China in 1996, which were usually associated with proventriculitis [45]. European QX like IBV strains are associated with nephropathogenicity and cystic oviducts [6,39]. In Europe, the nephropathogenicity of QX like IBV strains may have derived from the initial European nephropathogenic strains like B1648. By natural recombination events segments of the B1648 genome may have been transferred into genomes of NGA/A116E7/2006, UKr 27-11, ITA/90254/2005 and CK/SWE/0658946/10. The emergence or evolution of different coronaviral genotypes or strains by recombination events has been well documented in IBV and other coronaviruses [5,46]. Recombination analysis has suggested that the genetic recombination sites can be located in multiple genes [12]. Regions that have the highest occurrence of recombination were located on the parts of replicase transcriptase complex (NSP2, 3, 4, 6, 13 and 14) and spike protein. The rate of recombination may be one of the important mechanisms for generating genetic and antigenic diversity within IBV [5,46]. Accumulation of mutations and recombination events between the live vaccines and field strains likely produce novel variant strains or recombinant strains like, NGA/A116E7/2006 or Ukr27-11 [19]. These novel strains are known to cause disease epidemics in chickens and vaccination failure [12], and further studies are necessary to better understand the frequency of natural recombination events and which genes are preferentially involved in recombination.

Pairwise comparisons have shown that B1648 was closely related to pathogenic non-Mass type strains but not to Massachusetts type strains and vaccines. Based on the full-length genome, Nigerian reference IBADAN strain (NGA/A116E7/2006) was the closest relative with 91.6% nucleotide identity. Next closest were Ukrainian (UKr 27-11) and American (Gray and JMK) strains with 91.2% nucleotide identity. It is known that Gray is a nephropathogen and JMK is a respiratory pathogen, but the information on NGA/A116E7/2006 and UKr 27-11 strains about their tissue tropism is not available. Pairwise comparisons of 1a, Spike, M and accessory proteins (3a, 3b, 4b, 4c, 5a, 5b and 6b) have suggested that B1648 was most closely related to non-Mass type strains. However, based on 1b, E and N proteins B1648 was closely related to both Mass and non-Mass types strains. All these comparisons have implicated that the determinants of nephropathogenicity (B1648 strain) is most probably located on the 1a, spike, M and accessory proteins. Some authors have hypothesized that the pathogenicity determinants of IBV may be multi-genic, and associated outside the spike protein [47,48,49]. With the well-studied coronavirus, murine hepatitis virus (MHV), NSP1, Nsp3 and Nsp14 have been linked with virulence [50,51,52]. It is very well possible that the non-structural proteins (Nsp1 to Nsp11) encoded by ORF 1a are strong candidates for being involved in the nephropathogenicity. This will be investigated in the near future.

Classification of the IBV serotypes is mainly based on the variability of the spike protein or partial S1 fragment [3,19,39,53]. According to partial S1 gene analysis, Tunisian (TN20/00), Russian (IBV-27/99 and RF/06/2007) and Slovenian (SLO/266/05) strains were clustered together with B1648. This cluster is referred to as B1648 genotype. Moreover, TN20/00 was the closest among all the reported IBV strains with 97.4% nucleotide homology. Next closest were RF-27/99 (96.4%), RF/06/2007 (96.1%) and SLO/266/05 (89.4%) [18,20,22]. The partial S1 fragment analysis has revealed that the B1648 genotype is one of the important IBV genotypes, which has been circulating in Europe and North Africa for over three decades. Although the B1648 strain outbreak has occurred in 1984, its origin remains still unidentified. This raises the question, whether B1648 type has emerged from another animal species, like MERS-CoV of humans emerged from bats/camels [54,55]. The comparison with MERS-CoV is interesting because it is also associated with kidney problems in humans. However, the phylogenetic analysis, full-length nucleotide identities and recombination analysis has shown that B1648 is distinct from other known avian and mammalian coronaviruses, and provides no information on its origin (data not shown). Based on the danger of cross species jumps of coronaviruses, more epidemiologic and surveillance studies should be done on coronaviruses in species living in the wild. Gammacoronaviruses could be circulating asymptomatically in wild birds as reservoirs, before emerging as a novel pathogenic IBV strains in chickens [56,57]. In this context, efforts should be done to generate a database of full-length sequences of coronaviruses in wild animals e.g., wild migratory birds.

In summary, the present study has demonstrated that B1648 is a distinct strain setting it apart from all strains reported so far in Europe and other parts of the world. Partial S1 gene analysis has suggested that B1648 genotype or its variants has been circulating in Europe and North Africa for over three decades. The pathogenicity determinants of B1648 strain might be located on the 1a, spike, M and accessory proteins (3a, 3b, 4b, 4c, 5a, 5b and 6b). By reverse genetics the molecular basis of the nephropathogenicity of IBV strains will be elucidated.

## Supplementary Files

Supplementary File 1
