# Supplementary material for: Genetic Characterization of the Belgian Nephropathogenic Infectious Bronchitis Virus (NIBV) Reference Strain B1648
Source: Viruses. 2015 Aug 7;7(8):4488–506. doi: 10.3390/v7082827 (PMC4576188; doi:10.3390/v7082827)
Supplement: Supplementary File 1 [file viruses-07-02827-s001.pdf]

Supplementary Information

Table S1. Amino acid sequence identity (%) of B1648 polyprotein 1ab compared with relevant IBVs.

Strain	NSP 2	NSP 3	NSP 4	NSP 5	NSP 6	NSP 7	NSP 8	NSP 9	NSP 10	NSP 11	NSP 12	NSP 13	NSP 14	NSP 15	NSP 16
Beaudette	90.6	89.8	90.3	95.0	92.5	97.5	98.0	98.1	97.2	80.9	97.1	97.6	97.0	95.4	93.5
BJ	85.1	*80.6*	85.0	90.8	86.8	96.3	93.5	98.1	95.7	75.5	96.9	*95.9*	94.9	93.2	92.4
California99	92.1	88.5	92.3	96.3	93.3	98.8	96.5	98.1	97.2	86.0	97.8	99.2	96.4	95.4	94.2
Cal557_2003	91.1	87.1	92.5	96.7	93.3	97.5	98.0	99.1	97.2	86.0	97.7	**99.2**	97.8	95.1	93.5
Cal56b	92.1	89.0	91.8	98.4	93.6	97.5	95.0	99.1	96.5	80.9	97.6	99.0	96.4	95.4	93.8
SAIBK	*84.7*	87.5	91.8	93.6	90.3	97.5	*91.4*	97.2	97.9	80.9	96.5	97.3	96.4	92.3	91.3
TW2575/98	85.8	83.4	91.2	93.6	88.0	98.8	95.0	98.1	96.5	75.5	96.6	97.4	96.0	93.2	91.3
ArkDPI11	92.7	88.1	91.8	98.4	93.6	**100.0**	98.5	99.1	98.6	80.9	97.3	99.0	97.0	94.8	93.5
Strain_A2	85.1	80.7	*83.9*	*87.9*	*85.3*	95.0	93.0	98.1	*94.3*	*75.5*	*96.2*	97.4	*92.4*	*90.3*	90.6
CK/CH/LSD/05I	91.8	85.7	89.7	93.2	89.2	97.5	94.0	98.1	95.7	75.5	96.4	97.6	96.0	93.2	90.9
SC021202	84.7	87.7	92.3	94.3	90.7	97.5	96.0	99.1	96.5	75.5	96.5	96.7	97.4	91.6	90.9
H52	92.7	88.1	92.9	95.3	89.5	98.8	97.0	97.2	97.9	69.8	97.6	98.1	96.8	93.5	93.1
H120	92.7	88.1	92.9	96.0	89.5	97.5	98.5	98.1	97.9	86.0	**98.0**	98.8	97.8	95.7	93.5
Mass41_2006	92.3	89.9	91.4	98.0	93.6	98.8	98.0	98.1	97.9	86.0	97.5	98.5	97.6	95.7	93.5
Mass41_1985	89.1	90.2	89.0	95.0	93.3	98.8	97.0	97.2	97.9	69.8	97.5	98.3	97.0	93.2	94.5
Conn46_1996	91.4	91.0	89.5	98.4	93.6	100.0	98.0	**99.1**	96.5	86.0	97.3	99.0	96.6	95.1	94.5
ITA/90254/2005	94.5	92.2	94.4	95.0	95.4	98.8	99.0	98.1	97.9	80.9	98.0	98.3	96.6	93.2	*90.2*
NGA/A116E7/2006	92.4	91.8	**94.8**	97.4	95.8	97.5	**99.5**	98.1	97.9	86.0	97.9	98.6	97.0	93.9	93.1
Georgia_1998	92.7	87.8	92.9	**98.7**	93.6	100.0	98.5	99.1	97.9	86.0	97.6	99.0	97.0	95.1	93.5
Delaware_072	92.7	85.1	92.7	95.7	89.5	98.8	98.5	97.2	97.9	86.0	97.9	92.8	89.1	95.1	92.7
FL18288	91.6	90.8	90.1	98.4	93.6	100.0	97.5	99.1	96.5	86.0	97.6	99.0	95.1	95.4	94.2
Gray	90.5	**93.0**	89.7	97.7	94.0	100.0	98.0	98.1	97.2	80.9	97.7	98.8	97.6	95.4	93.5
Holte	92.3	91.0	90.5	97.7	93.6	98.8	98.5	96.3	96.5	86.0	97.2	98.8	98.0	95.1	93.1
Iowa_97	92.1	90.0	90.5	97.7	93.6	98.8	98.5	98.1	96.5	86.0	97.2	98.8	**98.0**	95.1	93.1
JMK	91.3	92.4	91.4	98.0	94.0	100.0	98.5	99.1	**98.6**	80.9	97.6	98.1	97.6	95.4	93.5
ck/CH/LDL/101212	92.4	87.9	92.5	96.0	89.5	98.8	98.5	97.2	97.9	86.0	97.9	98.8	97.8	95.7	93.5
CK/SWE/0658946/10	**95.0**	87.4	90.3	95.7	**95.8**	98.8	98.0	*69.1*	95.7	80.9	98.0	98.5	95.1	91.3	89.8
SNU8067	92.4	92.0	89.9	97.0	92.5	97.5	97.0	99.1	97.2	86.0	97.7	98.3	97.6	93.9	90.9

Table S1. *Cont.*

Strain	NSP 2	NSP 3	NSP 4	NSP 5	NSP 6	NSP 7	NSP 8	NSP 9	NSP 10	NSP 11	NSP 12	NSP 13	NSP 14	NSP 15	NSP 16
KM91	91.0	90.8	90.5	97.4	94.0	98.8	98.0	99.1	97.2	86.0	97.7	98.6	97.0	94.8	90.9
ck/CH/LDL/091022	85.4	84.8	85.0	88.6	86.8	96.3	93.5	99.1	94.3	75.5	96.6	97.8	97.4	93.2	90.9
ck/CH/LZJ/111113	85.1	85.5	85.2	89.3	87.2	96.3	93.0	98.1	95.7	75.5	96.3	97.1	97.0	92.6	93.5
Ck/CH/LGD/120723	85.3	84.0	86.4	89.7	86.5	*95.0*	93.0	98.1	94.3	75.5	96.4	97.6	96.8	92.9	91.3
ck/CH/LJL/111054	92.7	89.0	92.7	96.0	89.5	98.8	98.0	99.1	97.9	86.0	98.0	99.2	96.6	95.4	**94.5**
Ukr27-11	95.0	88.0	89.7	96.0	89.2	98.8	98.5	99.1	97.9	86.0	98.0	98.8	96.4	**96.1**	94.2
ck/CH/LDL/110931	92.7	88.1	92.9	96.0	89.5	97.5	98.5	99.1	97.9	**86.0**	98.0	98.8	97.8	95.7	93.1

Boldface indicates the highest. and italic. the lowest. amino acid sequence identity.

© 2015 by the authors; licensee MDPI, Basel, Switzerland. This article is an open access article distributed under the terms and conditions of the Creative Commons Attribution license (http://creativecommons.org/licenses/by/4.0/).
